# Resource‐Adapted Triage Strategies for Women Testing HPV Positive With Self‐Collected Vaginal Samples in Cameroon

**DOI:** 10.1002/ijc.70573

**Published:** 2026-06-18

**Authors:** Micol Murtas, Pierre Vassilakos, Ania Wisniak, Ketty Hu, Marc Arbyn, Gilles W. Tankeu Happi, Esther Abatsong, François Marcel Ndam Nsangou, Jean‐Christophe Tille, Essia Saiji, Bruno Kenfack, Patrick Petignat

**Affiliations:** ^1^ Gynecologic Division, Department of Pediatrics, Gynecology, and Obstetrics University Hospitals Geneva, University of Geneva Geneva Switzerland; ^2^ Geneva Foundation for Medical Education and Research Geneva Switzerland; ^3^ Institute of Global Health, Faculty of Medicine University of Geneva Geneva Switzerland; ^4^ Unit of Cancer Epidemiology Belgian Cancer Centre Brussels Belgium; ^5^ Department of Human Structure and Repair, Faculty of Medicine and Health Sciences University of Ghent Ghent Belgium; ^6^ Department of Gynaecology and Obstetrics Dschang Regional Annex Hospital Dschang Cameroon; ^7^ Division of Clinical Pathology Diagnostic Department Geneva University Hospitals Geneva Switzerland; ^8^ Department of Obstetrics and Gynaecology, Faculty of Medicine and Pharmaceutical Sciences University of Dschang Dschang Cameroon

**Keywords:** cervical cancer screening, extended genotyping, human papillomavirus, resource‐adapted strategies, risk‐based management

## Abstract

The World Health Organization (WHO) recommends HPV‐DNA testing as the primary screening method for cervical cancer in low‐ and middle‐income countries (LMICs), yet guidance on how to triage HPV‐positive women remains limited. The 2021 WHO guidelines for LMICs do not incorporate extended HPV genotyping, despite its potential to improve risk stratification and support scalable management strategies. This study evaluated screening strategies based on locally available resources for management of HPV‐positive women. We retrospectively analyzed data from the 3T study (Test, Triage, and Treat), conducted between 2018 and 2022, which included HPV self‐sampling and VIA triage among women aged 30–49 years. For all HPV‐positive participants, HPV testing (stratified into five genotype‐based risk groups), cytology, VIA, cervical‐biopsy, and endocervical‐brush histopathology were available. Thirty‐five screening and triage strategies integrating HPV extended genotyping alone or in combination with VIA or cytology were assessed using CIN3+ risk as a guiding principle. Among 868 HPV‐positive women, 9% had CIN3+ lesions. HPV types 31/33/35/52/58 were most prevalent, whereas HPV16 and HPV18/45 carried the highest CIN3+ risks. Strategies combining extended genotyping with cytology demonstrated the best balance of sensitivity and specificity. In settings without access to cytology or VIA, direct treatment of the eight highest‐risk HPV types, 16/18/45/31/33/35/52/58, showed the best balance between sensitivity and specificity. When cytology or VIA was available, immediate treatment for HPV16 and reflex triage, cytology (treating ≥ ASCUS lesions), or VIA for HPV18/45/31/33/35/52/58 showed the best trade‐off. The proposed screening strategies enable risk‐adapted triage and guide management decisions based on available resources.

## What's New?

Although human papillomavirus (HPV) testing is recommended for cervical cancer screening in low‐ and middle‐income countries, optimal management of HPV‐positive women remains undetermined, particularly in resource‐limited settings. Here, using data from the 3T study involving women in Cameroon, the authors evaluated the ability of extended HPV genotyping, visual inspection, and cytology to guide triage and treatment decisions. Analyses show that about 9 percent of HPV‐positive women had high‐grade precancerous lesions. Risk of lesions was highest for HPV16‐ or HPV18/45‐positive women. Strategies combining genotyping with cytology provided optimal guidance, though extended genotyping could inform triage when other approaches are unavailable.

## Graphical abstract

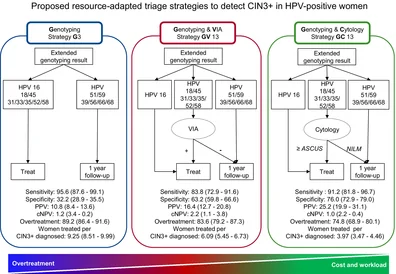

**Abbreviations**
AI: artificial intelligence
ASC‐US+: atypical squamous cells of undetermined significance or worse
CC: cervical cancer
CI: confidence interval
CIN2+: cervical intraepithelial neoplasia grade 2 or worse
CIN3+: cervical intraepithelial neoplasia grade 3 or worse
cNPV: complementary negative predictive value
DNA: deoxyribonucleic acid
ECB: endocervical brushing
HIV: human immunodeficiency virus
HPV: human papillomavirus
IARC: lnternational Agency for Research on Cancer
LLETZ: large loop excision of the transformation zone
LMICs: low‐ and middle‐income countries
mPPV: marginal positive predictive value
PPV: positive predictive value
VIA: visual inspection with acetic acid
WHO: World Health Organization

## 1 Introduction

Cervical cancer (CC) remains a significant public health challenge, particularly in low‐ and middle‐income countries (LMICs), where the disease burden is disproportionately high [1]. The established causal link between persistent infection with 13 oncogenic human papillomavirus (HPV) types and nearly all cervical precancers and cancers has led to the development of molecular HPV detection tests [2], now recommended for primary screening [3]. Moreover, the implementation of sensitive point‐of‐care HPV detection methods using self‐obtained vaginal samples has increased screening coverage, particularly in communities with limited access to healthcare facilities, owing to the convenience, cultural acceptability [4], and greater cost‐effectiveness of self‐obtained cervical samples compared with those of clinician‐collected samples [5]. However, clinical management of women who test positive for HPV remains debatable in low‐resource settings, as it depends on the available triage options. Strategies must weigh the benefit of CC prevention against the risks of unnecessary procedures and overtreatment, which may lead to increased costs, emotional distress, and potential clinical complications [6]. HPV genotyping is a promising type of triage because oncogenic HPV types vary widely in carcinogenicity. The International Agency for Research on Cancer (IARC) has established a hierarchy in four risk categories worldwide: HPV 16 (64% of cancers), followed by HPV 18 (17% of cancers); HPV 31, 33, 45, 52 and 58 (15%–20% of cancers); and HPV 35, 59, 39, 56, 51, 68, 73, 26, 69, and 82 (5% of cancers) [7]. Although most HPV types carry similar cancer risks across regions, exceptions may exist. Certain types, such as HPV 35, appear to be more prevalent and associated with higher cancer risk in specific populations, particularly among women of African ancestry [8]. A point‐of‐care assay stratifying HPV types according to their oncogenic risk has already been proposed for use in low‐income settings to guide triage in the general population [9] and in women living with human immunodeficiency virus (HIV) [10]. Three triage options involving genotype stratification can be considered, based on available resources and health system capacity: (i) genotyping alone; (ii) genotyping followed by visual inspection with acetic acid (VIA); or (iii) genotyping followed by cytology.

This study aimed to compare the performance of single and combined triage strategies, based on HPV DNA genotyping alone, or in combination with VIA and cytology, for managing patients with HPV. These findings aim to guide the selection of effective and context‐appropriate triage strategies within screen‐triage‐treat approaches.

## 2 Methods

### 2.1 Study Design

We conducted a retrospective analysis using the 3T (Test‐Triage‐Treat) study data, a prospective implementation study conducted at Dschang Regional Annex Hospital between 2018 and 2022. Overall, 4451 women aged 30–49 years were enrolled. Exclusion criteria comprised pregnancy, hysterectomy, severe medical conditions, and any factor that could impair cervical visualization during screening (e.g., metrorrhagia) [11].

HPV status was assessed using a point‐of‐care assay (GeneXpert technology, Cepheid, Sunnyvale, CA) on self‐collected vaginal samples. The test detects 14 carcinogenic genotypes categorized into five hierarchical carcinogenic risk groups: Channel 1 (HPV16), Channel 2 (HPV18/45), Channel 3 (HPV31/33/35/52/58), Channel 4 (HPV51/59), and Channel 5 (HPV39/56/66/68). All women testing positive for HPV underwent a standardized clinical assessment. Cervical samples were collected for liquid‐based cytology preparations. Women were then triaged using VIA ABCD criteria [12], assisted by smartphone imaging. Cervical biopsies and endocervical brushings (ECB) were performed following VIA assessment: targeted biopsies were obtained from VIA‐visible lesions, and in the absence of visible lesions, a random biopsy was taken from the 6 o'clock position of the transformation zone. Biopsies were performed prior to treatment and could induce minor bleeding; however, this was generally minimal and easily controlled (e.g., compression or silver nitrate) and did not affect treatment feasibility or safety.

Women with a positive VIA result were immediately treated with thermal ablation if eligible or referred for other treatment (i.e., LLETZ or multimodal therapy) if ineligible. Women were considered eligible for thermal ablation if the probe could cover the entire transformation zone. Cytology and genotyping results did not influence treatment decisions, which were based only on VIA findings (Figure S1).

Histological results from biopsies and ECB were obtained for all HPV‐positive women and used as the reference standards. Histologic and cytologic analyses were initially planned at Yaoundé Gyneco‐Obstetric and Pediatric Hospital; however, with the laboratory's relocation, the diagnoses were performed at the Pathology Division of Geneva University Hospitals, Switzerland. Data were stored in the SecuTrial electronic database.

### 2.2 Included Data

We included all participants with complete histological results from their first visit. In cases of multiple HPV infections, only the highest‐risk genotype was considered for analysis, based on the hierarchical risk classification of the GeneXpert assay [13]. Namely, if a participant had channels 1 and 3, we only considered channel 1 in the analysis. Histology was classified as positive if cervical intraepithelial neoplasia grade 3 or worse (CIN3+) was identified in either biopsy or ECB. Cytology was classified as positive for atypical squamous cells of undetermined significance or worse (ASC‐US+).

### 2.3 Strategy Design

We designed 35 distinct screening and management strategies, adaptable to different settings according to available resources and health system capacity. These strategies incorporate genotyping (G), VIA (V), and cytology (C), and are grouped into four patient management categories:

1. In the first category (G1–G5), patients are treated based on their HPV genotype only; those not treated are advised to undergo follow‐up (e.g., after 1 year) to check for HPV persistence (Table 1). Namely, in strategy G3, patients with HPV genotypes of channels 1, 2, and 3 are treated directly, while those with channels 4 and 5 are not treated and are monitored until HPV clearance.
2. In the second category, patients with certain genotypes undergo VIA (GV1–GV5) or cytology (GC1–GC5) triage and receive treatment if positive; those not triaged are advised to undergo monitoring for HPV persistence. For example, in strategy GV4, patients with HPV genotypes of channels 1 and 2 receive VIA triage (and are treated if VIA positive), while those with channels 3, 4, and 5 are not triaged and simply monitored for HPV persistence.
3. In the third category (GV6–GV9 and GC6–GC9), all patients are either directly treated or triaged by VIA or cytology, depending on their HPV genotype; no patient is simply monitored for persistence without treatment or triage. For example, patients with HPV genotypes of channels 1 and 2 are treated directly in strategy GC7, while those with channels 3, 4, and 5 undergo cytology triage (and are treated if ASCUS+).
4. In the fourth category (GV10–GV15 and GC10–GC15), management varies by HPV genotype; some patients directly receive treatment based on genotyping alone, others undergo VIA or cytology triage, and others are only advised to undergo follow‐up for HPV pe rsistence. For example, in strategy GV13, patients with a channel 1 are treated directly, those with channels 2 or 3 are triaged by VIA (and treated if positive), while those with a channel 5 are only monitored for HPV persistence (with no treatment or triage).

**TABLE 1:** Detailed explanation of the design of strategies.

	Direct treatment	Triage	Direct follow‐up at 1 year
Genotyping			
G 1^a^	1/2/3/4/5	—	—
G 2	1/2/3/4	—	5
G 3	1/2/3	—	4/5
G 4	1/2	—	3/4/5
G 5	1	—	2/3/4/5
Genotyping with triage by VIA			
GV 1^a^	—	1/2/3/4/5	—
GV 2	—	1/2/3/4	5
GV 3	—	1/2/3	4/5
GV 4	—	1/2	3/4/5
GV 5	—	1	2/3/4/5
GV 6	1	2/3/4/5	—
GV 7^a^	1/2	3/4/5	—
GV 8	1/2/3	4/5	—
GV 9	1/2/3/4	5	—
GV 10	1	2/3/4	5
GV 11	1/2	3/4	5
GV 12	1/2/3	4	5
GV 13	1	2/3	4/5
GV 14	1/2	3	4/5
GV 15	1	2	3/4/5
Genotyping with triage by cytology (≥ ASCUS)			
GC 1^a^	—	1/2/3/4/5	—
GC 2	—	1/2/3/4	5
GC 3	—	1/2/3	4/5
GC 4	—	1/2	3/4/5
GC 5	—	1	2/3/4/5
GC 6	1	2/3/4/5	—
GC 7	1/2	3/4/5	—
GC 8	1/2/3	4/5	—
GC 9	1/2/3/4	5	—
GC 10	1	2/3/4	5
GC 11	1/2	3/4	5
GC 12	1/2/3	4	5
GC 13	1	2/3	4/5
GC 14	1/2	3	4/5
GC 15	1	2	3/4/5

*Note:* “/” indicates “or”; Channel 1 = HPV16, Channel 2 = HPV18/45, Channel 3 = HPV31/33/35/52/58, Channel 4 = HPV51/59, Channel 5 = HPV39/56/66/68; HPV‐positive women are followed up at 1 year.

Abbreviations: ASC‐US = atypical squamous cells of undetermined significance or worse; VIA = visual inspection with acetic acid.

^a^ Strategies recommended in the 2021 WHO guidelines.

The 2021 WHO guidelines recommend four of these strategies: G1, GV1, GV7, and GC1 (Table 1).

### 2.4 Statistical Analysis

For each channel, we calculated the absolute risk of CIN2+ and CIN3+ lesions.

For each strategy, we determined sensitivity, specificity, positive predictive value (PPV), complementary negative predictive value (cNPV), overtreatment rate (1–PPV), the Youden index (YI), and number of women treated per CIN3+ diagnosis. While formal statistical comparisons between strategies were not performed, we visualized performance by plotting sensitivity against 1–specificity, with associated 95% confidence intervals (CI). Predefined thresholds were used to identify clinically acceptable strategies, with sensitivity > 85%, specificity > 80%, PPV > 20%, complement of the negative predictive value (cNPV) < 5%, and overtreatment < 60%. These criteria were selected a priori to reflect a pragmatic balance between maximizing detection of CIN3+ lesions and minimizing overtreatment, in line with the programmatic principles of CC screening in low‐resource settings as outlined by the World Health Organization. We conducted an incremental benefit‐harm analysis analogous to a cost‐effectiveness analysis to evaluate the trade‐off between benefits (detection of CIN3+) and harms (potential overtreatment following a positive result). The number of individuals testing positive under each strategy served as a proxy for cost or harm, while the number of CIN3+ cases detected represented effectiveness or benefit. Strategies were ranked by increasing positivity rates, and the marginal positive predictive value (mPPV) was defined as the additional number of CIN 2+ or CIN3+ cases detected per additional positive result (i.e., treated patient) between two non‐dominated strategies. The efficient frontier was defined as the set of non‐dominated strategies offering an optimal balance between detection and overtreatment. To compare two non‐dominated strategies, I and II, the mPPV was calculated as follows: mPPV = (Number of CIN3+ cases detected with strategy II—Number of CIN3+ cases detected with strategy I) / (Number of women positive under strategy II—Number of women positive under strategy I). This metric reflects the additional CIN3+ detection yield per additional woman treated when moving from strategy I to strategy II [14, 15]. For a limited number of pairwise comparisons between selected strategies informed by the main analysis, sensitivities and specificities were compared using McNemar's test for paired proportions. Sensitivity was assessed among women with CIN3+, and specificity among women without CIN3+. These analyses were exploratory and restricted to a small number of comparisons to limit multiplicity and preserve interpretability. All analyses were performed for a CIN2+ threshold. Analyses were conducted using R version 4.3.2.

## 3 Results

**FIGURE 1:**
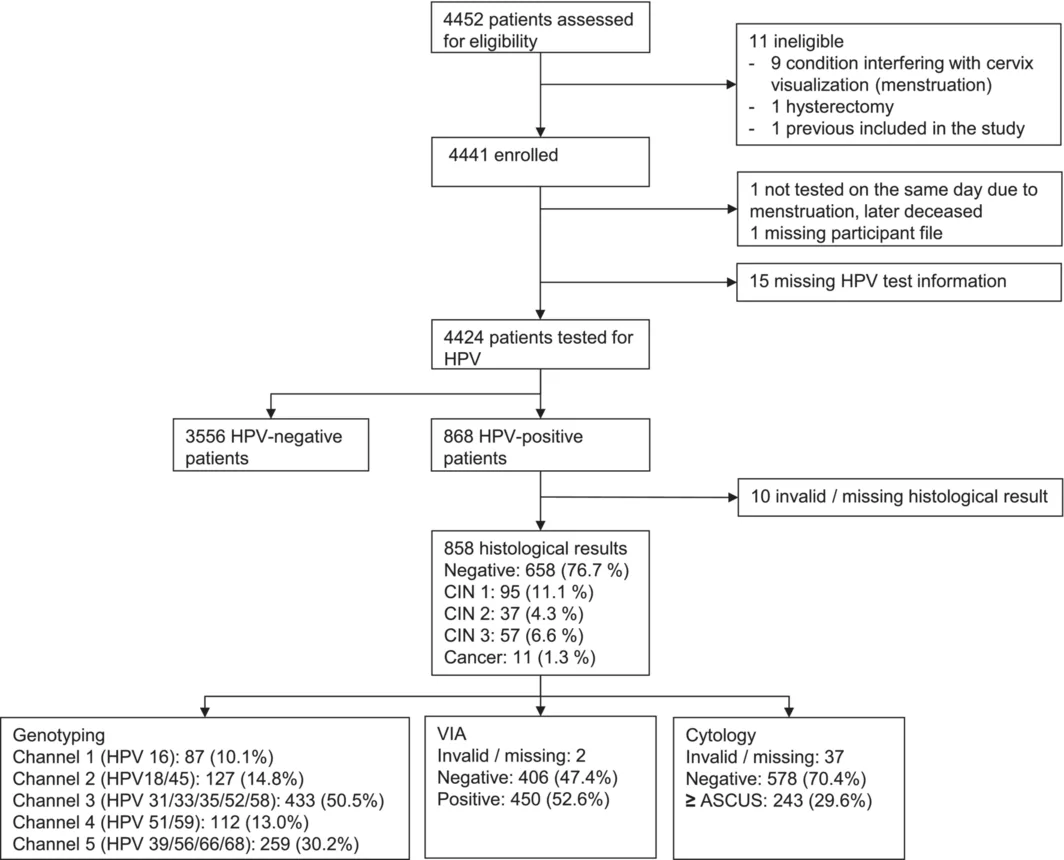
Flowchart illustrating the study population and available data. For HPV types, all positive channels are shown without any specific order of severity. ASC‐US = atypical squamous cells of undetermined significance or worse.

Among the 4452 participants assessed for eligibility, 11 were deemed ineligible (Figure 1); 868 (19.6%) of the 4424 remaining women were HPV‐positive. Valid histological results were available for 858 women (98.8%), among whom 105 (12.2%) had CIN2+ lesions, 68 (7.9%) CIN3+ lesions, and 11 (1.3%) were diagnosed with CC. VIA was positive in 450 women (52.6%), while cytology showed ASCUS or worse in 243 cases (28.3%). Two women (0.2%) had missing or invalid VIA results, and 37 (4.3%) had invalid cytology results. All patients had valid HPV genotyping results. Channel 3 was the most frequent genotype (433 women, 50.5%), followed by channels 5 (*n* = 259, 30.2%), 2 (*n* = 127, 14.8%), 4 (*n* = 112, 13.0%), and 1 (*n* = 87, 10.1%). Among women with HPV, 139 had multiple infections; in the subset with valid histology, 136 had multiple infections. The median age was 39 years in the HPV‐positive group and 40 years in the HPV‐negative group (Table S1). Among 198 patients with HIV, 64 (32.3%) tested positive for HPV (Table S2). Sociodemographic characteristics of participants are shown in Table S1.

### 3.1 Absolute Risk by HPV Genotype

**FIGURE 2:**
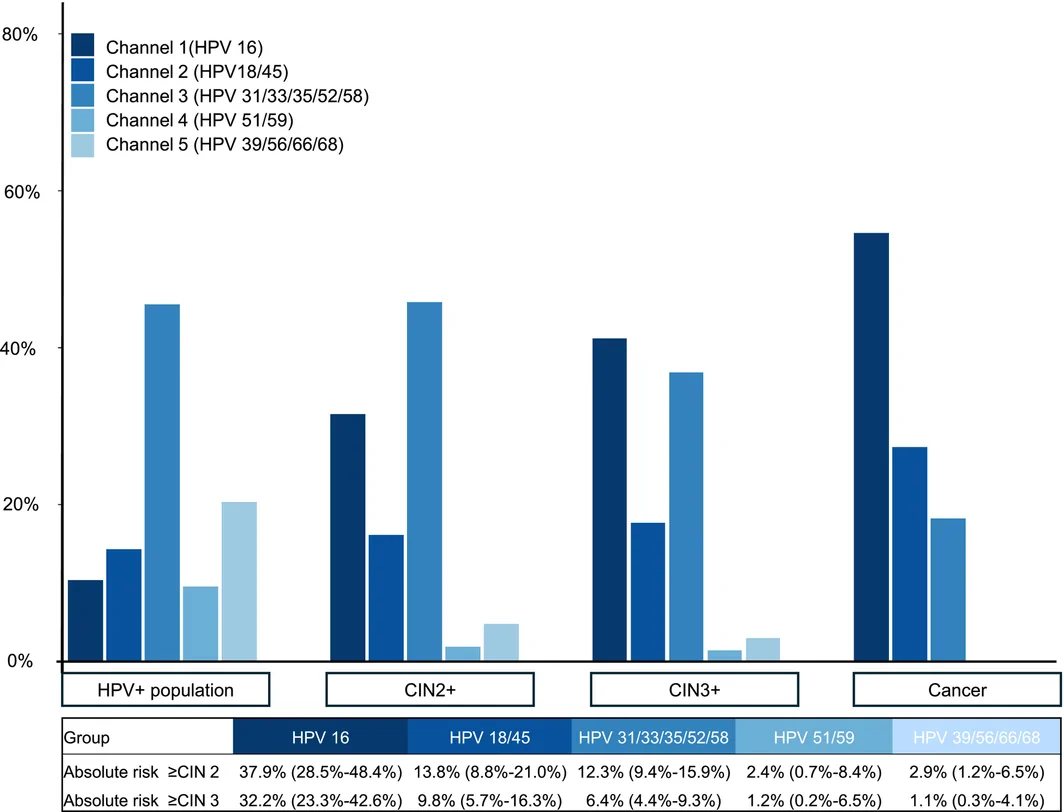
Prevalence of HPV type according to histological result and absolute risk of CIN 2+ and CIN 3+ lesions for each HPV channel. CIN2+, cervical intraepithelial neoplasia grade two or worse; CIN3+, cervical intraepithelial neoplasia grade three or worse; In cases of multiple HPV infections, only the highest‐risk genotype was considered, based on the hierarchical classification of the GeneXpert assay (channel 1, then 2, 3, 4, and 5).

CIN2+ lesions were most frequently detected in channel 3, followed by channels 1 and 2, whereas CIN3+ lesions were most frequently detected in channel 1, followed by channels 3 and 2 (Figure 2). Among cancer cases, 6 (54.5%) were linked to channel 1, 3 (27.3%) to channel 2, and 2 (18.2%) to channel 3. No cancer cases were detected in channels 4 or 5 (Figure 2 and Table S3). The absolute risk of CIN3+ was 32.2% for channel 1, 9.8% for channel 2, 6.4% for channel 3, 1.2% for channel 4, and 1.1% for channel 5 (Figure 2).

### 3.2 Strategy Comparison

**FIGURE 3:**
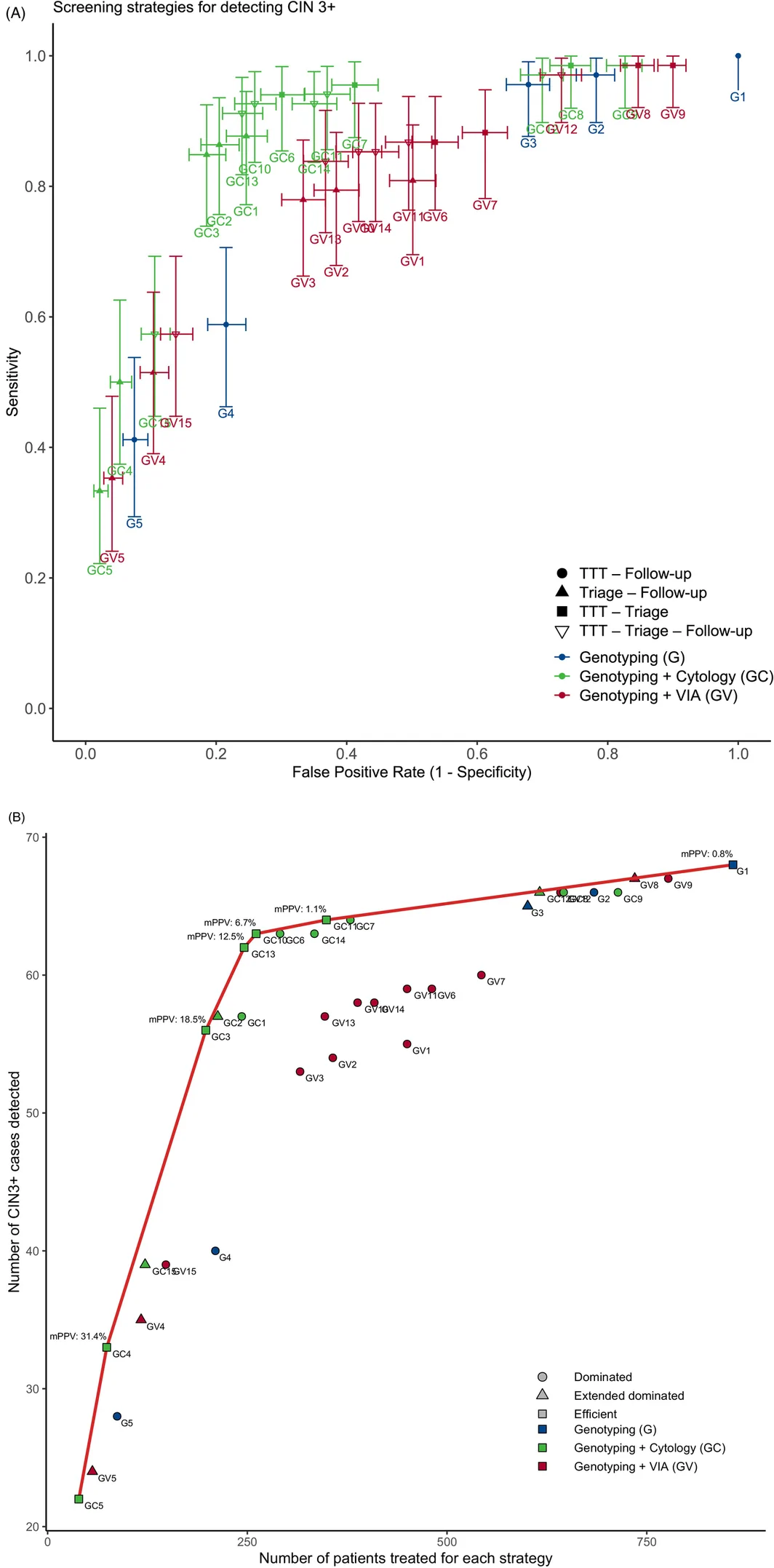
Receiver operating characteristic (ROC) plot (A) and efficient frontier (B) among the 35 triage strategies (A) Receiver operating characteristic (ROC) plot of the 35 strategies for detecting cervical intraepithelial neoplasia grade 3 or worse (CIN3+). Strategies combining HPV genotyping, VIA, and cytology are organized around direct treatment, triage, or follow‐up. (B) Overview of the efficient frontier among the 35 triage strategies. Each strategy is represented by the number of patients testing positive, plotted against the number of CIN3+ cases detected. The red curve highlights the efficient frontier, with annotated points indicating the most efficient strategies and their corresponding marginal positive predictive values (mPPV).

Table 2 displays the diagnostic performance of the 35 strategies for a CIN 3+ threshold. Excluding G1, which by design has a 100% sensitivity and 0% specificity, GV8 and GV9 demonstrated the highest sensitivity (98.5% [95% CI: 92.1–100]) and lowest specificity (15.3% [12.9–18.0] and 10.0% [8.0–12.3], respectively). GC5 had the highest specificity (97.8% [96.6–98.7]) but the lowest sensitivity (33.3% [22.2–46.0]). No strategy fulfilled all five predefined performance targets (sensitivity > 85%, specificity > 80%, PPV > 20%, cNPV < 5%, overtreatment < 60%) (Table 2). Diagnostic performance of all strategies for CIN3+ detection is shown in Figure 3A. Results of the sensitivity analysis using CIN2+ as the threshold are presented in the Appendix, Table S4, and Figure S2. Strategies, PPV, cNPV, and overtreatment are plotted for CIN2+ and CIN3+ in Figure S3.

**TABLE 2:** Sensitivity, specificity, PPV, NPV, women treated per CIN3+ diagnosed, and overtreatment of triage strategies.

	Sensitivity	Specificity	PPV	cNPV	Overtreatment in HPV + population	Women treated per CIN3+ diagnosed	Overtreatment in screened population
G 1^a^	100.0 (94.7–100.0)	0.0 (0.0–0.5)	7.9 (6.2–9.9)	0.0 (0.4–0.0)	92.1 (90.1–93.8)	12.62 (11.77–13.46)	17.9 (16.8–19.1)
G 2	97.1 (89.8–99.6)	21.8 (18.9–24.8)	9.6 (7.5–12.1)	1.1 (4.1–0.1)	90.4 (87.9–92.5)	10.36 (9.59–11.14)	14.0 (13.0–15.1)
G 3	95.6 (87.6–99.1)	32.2 (28.9–35.5)	10.8 (8.4–13.6)	1.2 (3.4–0.2)	89.2 (86.4–91.6)	9.25 (8.51–9.99)	12.1 (11.2–13.2)
G 4	58.8 (46.2–70.6)	78.5 (75.4–81.3)	19.0 (14.0–25.0)	4.3 (6.2–2.9)	81.0 (75.0–86.0)	5.25 (4.54–5.96)	3.9 (3.3–4.5)
G 5	41.2 (29.4–53.8)	92.5 (90.5–94.3)	32.2 (22.6–43.1)	5.2 (7.0–3.7)	67.8 (56.9–77.4)	3.11 (2.45–3.76)	1.3 (1.0–1.7)
Genotyping with triage by VIA							
GV 1^a^	80.9 (69.5–89.4)	49.9 (46.3–53.4)	12.2 (9.3–15.6)	3.2 (1.7–5.4)	87.8 (84.4–90.7)	8.18 (7.43–8.94)	8.9 (8.1–9.8)
GV 2	79.4 (67.9–88.3)	61.6 (58.1–65.0)	15.1 (11.6–19.3)	2.8 (1.5–4.7)	84.9 (80.7–88.4)	6.61 (3.23–4.24)	6.9 (6.1–7.7)
GV 3	77.9 (66.2–87.1)	66.7 (63.3–70.0)	16.8 (12.8–21.4)	2.8 (1.6–4.5)	83.2 (78.6–87.2)	5.96 (5.30–6.62)	6.0 (5.3–6.7)
GV 4	51.5 (39.0–63.8)	89.6 (87.3–91.6)	29.9 (21.8–39.1)	4.5 (3.1–6.2)	70.1 (60.9–78.2)	3.34 (2.74–3.95)	1.9 (1.5–2.3)
GV 5	35.3 (24.1–47.8)	95.9 (94.3–97.2)	42.9 (29.7–56.8)	5.5 (4.0–7.3)	57.1 (43.2–70.3)	2.33 (1.72–2.94)	0.7 (0.5–1.0)
GV 6	86.8 (76.4–93.8)	46.4 (42.9–50.0)	12.3 (9.5–15.5)	2.4 (1.1–4.5)	87.7 (84.5–90.5)	8.15 (7.42–8.88)	9.6 (8.7–10.5)
GV 7^a^	88.2 (78.1–94.8)	38.8 (35.4–42.3)	11.0 (8.5–14.0)	2.5 (1.1–5.0)	89.0 (86.0–91.5)	9.05 (8.29–9.81)	10.9 (10.0–11.9)
GV 8	98.5 (92.1–100.0)	15.3 (12.9–18.0)	9.1 (7.1–11.4)	0.8 (0.0–4.5)	90.9 (88.6–92.9)	10.97 (10.18–11.76)	15.1 (14.1–16.2)
GV 9	98.5 (92.1–100.0)	10.0 (8.0–12.3)	8.6 (6.7–10.8)	1.2 (0.0–6.8)	91.4 (89.2–93.3)	11.60 (10.78–12.41)	16.1 (15.0–17.2)
GV 10	85.3 (74.6–92.7)	58.2 (54.6–61.6)	14.9 (11.6–18.9)	2.1 (1.0–3.9)	85.1 (81.1–88.4)	6.69 (6.02–7.36)	7.5 (6.7–8.3)
GV 11	86.8 (76.4–93.8)	50.5 (47.0–54.0)	13.1 (10.1–16.6)	2.2 (1.0–4.1)	86.9 (83.4–89.9)	7.63 (6.92–8.33)	8.9 (8.0–9.7)
GV 12	97.1 (89.8–99.6)	27.1 (24.0–30.3)	10.3 (8.0–12.9)	0.9 (0.1–3.3)	89.7 (87.1–92.0)	9.73 (8.97–10.48)	13.0 (12.1–14.1)
GV 13	83.8 (72.9–91.6)	63.2 (59.8–66.6)	16.4 (12.7–20.8)	2.2 (1.1–3.8)	83.6 (79.2–87.3)	6.09 (5.45–6.73)	6.6 (5.9–7.4)
GV 14	85.3 (74.6–92.7)	55.6 (52.0–59.1)	14.2 (10.9–17.9)	2.2 (1.1–4.1)	85.8 (82.1–89.1)	7.05 (6.37–7.74)	8.0 (7.2–8.8)
GV 15	57.4 (44.8–69.3)	86.2 (83.6–88.5)	26.4 (19.5–34.2)	4.1 (2.8–5.8)	73.6 (65.8–80.5)	3.79 (3.18–4.41)	2.5 (2.0–3.0)
Genotyping with triage by cytology (≥ASCUS)							
GC 1^a^	87.7 (77.2–94.5)	75.4 (72.2–78.4)	23.5 (18.3–29.3)	1.4 (2.7–0.6)	76.5 (70.7–81.7)	4.26 (3.73–4.80)	4.2 (3.6–4.9)
GC 2	86.4 (75.7–93.6)	79.5 (76.5–82.3)	26.8 (20.9–33.2)	1.5 (2.8–0.7)	73.2 (66.8–79.1)	3.74 (3.23–4.24)	3.5 (3.0–4.1)
GC 3	84.8 (73.9–92.5)	81.5 (78.5–84.2)	28.3 (22.1–35.1)	1.6 (2.9–0.8)	71.7 (64.9–77.9)	3.54 (3.04–4.03)	3.2 (2.7–3.8)
GC 4	50.0 (37.4–62.6)	94.8 (93.0–96.2)	44.6 (33.0–56.6)	4.3 (5.9–3.0)	55.4 (43.4–67.0)	2.24 (1.73–2.75)	0.9 (0.7–1.3)
GC 5	33.3 (22.2–46.0)	97.8 (96.6–98.7)	56.4 (39.6–72.2)	5.4 (7.2–3.9)	43.6 (27.8–60.4)	1.77 (1.22–2.33)	0.4 (0.2–0.6)
GC 6	94.0 (85.4–98.3)	69.9 (66.5–73.2)	21.6 (17.1–26.8)	0.7 (1.9–0.2)	78.4 (73.2–82.9)	4.62 (4.09–5.15)	5.2 (4.5–5.9)
GC 7	95.5 (87.5–99.1)	58.8 (55.2–62.3)	16.9 (13.3–21.0)	0.7 (1.9–0.1)	83.1 (79.0–86.7)	5.92 (5.33–6.52)	7.1 (6.4–7.9)
GC 8	98.5 (92.0–100.0)	25.6 (22.6–28.9)	10.2 (8.0–12.8)	0.5 (2.7–0.0)	89.8 (87.2–92.0)	9.79 (9.03–10.54)	13.1 (12.2–14.2)
GC 9	98.5 (92.0–100.0)	17.3 (14.8–20.2)	9.2 (7.2–11.6)	0.7 (4.0–0.0)	90.8 (88.4–92.8)	10.82 (10.02–11.61)	14.7 (13.7–15.8)
GC 10	92.6 (83.7–97.6)	74.1 (70.8–77.2)	24.1 (19.1–29.8)	0.9 (2.0–0.3)	75.9 (70.2–80.9)	4.14 (3.64–4.65)	4.5 (3.9–5.1)
GC 11	94.1 (85.6–98.4)	63.0 (59.5–66.4)	18.3 (14.4–22.8)	0.8 (2.1–0.2)	81.7 (77.2–85.6)	5.45 (4.88–6.03)	6.5 (5.8–7.2)
GC 12	97.1 (89.8–99.6)	30.0 (26.8–33.4)	10.7 (8.4–13.4)	0.8 (3.0–0.1)	89.3 (86.6–91.6)	9.33 (8.60–10.07)	12.5 (11.5–13.5)
GC 13	91.2 (81.8–96.7)	76.0 (72.9–79.0)	25.2 (19.9–31.1)	1.0 (2.2–0.4)	74.8 (68.9–80.1)	3.97 (3.47–4.46)	4.2 (3.6–4.8)
GC 14	92.6 (83.7–97.6)	65.0 (61.5–68.3)	18.9 (14.8–23.5)	1.0 (2.3–0.3)	81.1 (76.5–85.2)	5.30 (4.73–5.87)	6.1 (5.5–6.9)
GC 15	57.4 (44.8–69.3)	89.4 (87.0–91.5)	32.0 (23.8–41.0)	4.0 (5.7–2.7)	68.0 (59.0–76.2)	3.13 (2.57–3.68)	1.9 (1.5–2.3)

*Note:* Channel 1 = HPV16, Channel 2 = HPV18/45, Channel 3 = HPV31/33/35/52/58, Channel 4 = HPV51/59, Channel 5 = HPV39/56/66/68.

Abbreviations: ASC‐US, atypical squamous cells of undetermined significance or worse; CIN3+, cervical intraepithelial neoplasia grade three or worse; cNPV, complementary negative predictive value; PPV, positive predictive value; VIA, Visual inspection assessment.

^a^ Recommended strategies in the 2021 WHO report.

**FIGURE 4:**
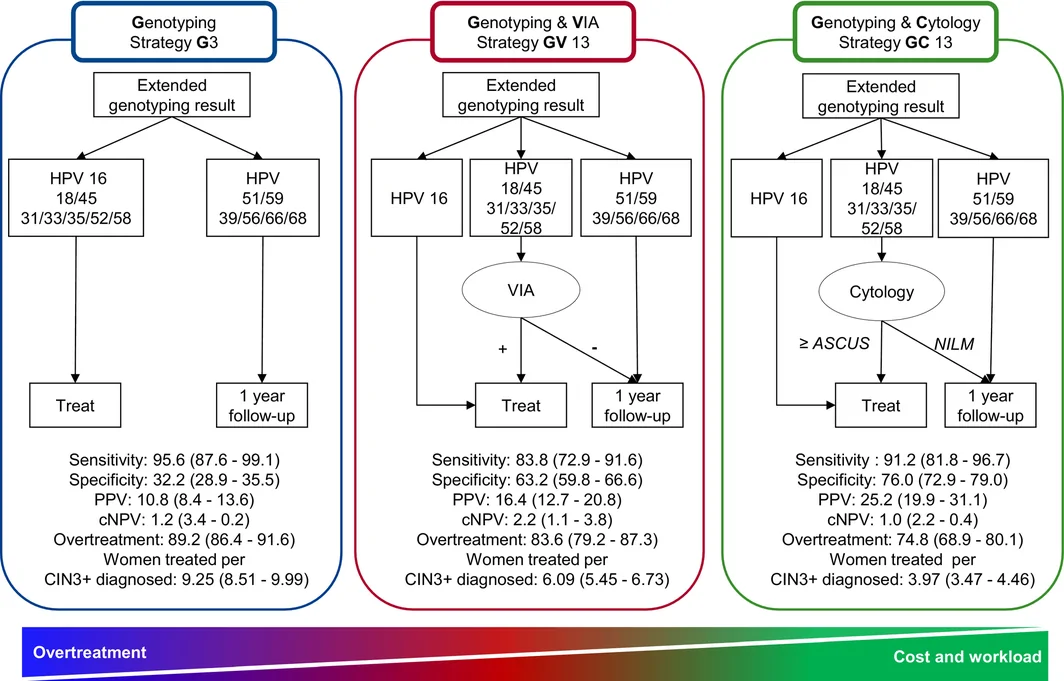
Preferred strategies based on available resources for CIN3+ PPV: Positive predictive value; cNPV: Complementary negative predictive value; VIA, Visual inspection assessment; ASC‐US, atypical squamous cells of undetermined significance or worse; CIN3+, cervical intraepithelial neoplasia grade three or worse.

In the first category (G1–G5), strategies only used genotyping. Strategy G3, directly treating channels 1–3 and following up channels 4 and 5, demonstrated the highest YI (0.27, 95% CI 0.17–0.35), with a sensitivity of 95.6% (87.6–99.1) and specificity of 32.2% (95% CI 28.9–35.5), treating 9.3 (8.5–10.0) women per CIN 3+ diagnosed (Figure 4).

In the second category (GV1–GV5 / GC1–GC5), the WHO‐recommended strategy GV1 (VIA triage of all HPV‐positive women) demonstrated 80.9% (69.5–89.4) sensitivity and 49.9% (46.3–53.4) specificity, with an overtreatment rate of 87.8% (84.4–90.7) among women with HPV. GC1, another WHO‐recommended strategy in which all women with HPV are triaged with cytology, showed a sensitivity of 87.7% (77.2–94.5) and a specificity of 75.4% (72.2–78.4). The overtreatment rate was 76.5% (70.7–81.7), with 4.3 (3.7–4.8) women treated per CIN3+ diagnosis.

In the third category (GV6–GV9/GC6–GC9), GC8, GC9, GV8, and GV9 showed a marked loss in specificity with minimal‐to‐no gain in sensitivity when compared to G3, offering limited diagnostic advantage.

In the fourth category (GV10–GV15/GC10–GC15), GV13 demonstrated the highest YI of 0.47 (0.33–0.58), with a sensitivity of 83.8% (72.9–91.6), specificity of 63.2% (59.8–66.6), PPV of 16.4% (12.7–20.8), and a cNPV of 2.2% (1.1–3.8), with 6.09 (5.45–6.73) women treated per CIN3+ diagnosed. Compared to GV1, GV13 had higher sensitivity and specificity and required fewer women (6.1 vs. 8.2) to be treated per CIN3+ case (Table 2). Among cytology triage strategies, GC13 demonstrated the highest YI of 0.67 (0.55–0.76), with a sensitivity of 91.2% (81.8–96.7), specificity of 76.0% (72.9–79.0), PPV of 25.2% (19.9–31.1), and overtreatment of 74.8% (68.9–80.1). cNPV was 1.0% (2.2–0.4), with 3.97 (3.5–4.5) women treated per CIN3+ diagnosed (Figure 4).

### 3.3 Comparison Between Strategies Using Cytology (GC1–GC15) and VIA (GV1‐15)

Overall, strategies incorporating cytology showed higher sensitivity compared to those using VIA, with differences ranging from—2.0% (GC5 vs. GV5) to +7.4% (GC13 vs. GV13) for sensitivity and 1.9% (GC5 vs. G5) to +25.5% (GC1 vs. GV1) for specificity (Table 2 and Figure 3A). VIA outperformed cytology in sensitivity for GC4 (−1.5%) and GC5 (−2.0%), whereas no difference was observed for GC8, GC9, GC12, and GC15. For PPV, cytology systematically outperformed VIA, with gains ranging from 0.4% (GC12 vs. GV12) to 14.7% (GC4 vs. GV4). Triaging all patients with HPV using cytology (GC1) instead of VIA (GV1) led to an increase of +6.8% in sensitivity, +25.5% in specificity, and + 11.3% for PPV. Figure S4 illustrates the best trade‐off among the strategy groups for detecting CIN2+ lesions. Table S5 shows pairwise comparisons between selected strategies. Sensitivity did not differ significantly between most strategies, whereas specificity was higher for cytology‐based strategies compared with VIA‐based or genotyping‐only strategies.

### 3.4 Harm–Benefit Analysis

Figure 3B presents the efficient frontier for the 35 evaluated screening strategies, plotting the number of patients treated against the number of CIN3+ cases detected. Strategies along this frontier reflect the optimal balance between diagnostic advantage and overtreatment. Strategy GC13 detected 62 CIN3+ cases while treating 246 women (PPV 25.2 [19.9–31.1]), representing the most efficient cytology‐based approach. Strategy G3 identified 65 CIN3+ cases for 601 women treated (PPV 10.8 [8.4–13.6]), combining high sensitivity with moderate overtreatment. GV strategies demonstrated more heterogeneous performance. GV13 detected 57 CIN3+ cases for 347 women treated, with (PPV 16.4 [12.7–20.8]), while GV1 required treatment of 450 women for 55 CIN3+ cases, with PPV 12.2 (9.3–15.6). GC strategies were closer to the efficiency frontier, whereas genotyping‐only strategies generally required treating more women per lesion detected.

## 4 Discussion

We comprehensively evaluated 35 CC screening strategies using HPV genotyping, either alone (G1–G5) or combined with triage (VIA or cytology GV/GC1–15), across four management categories. These strategies support context‐specific decision‐making in countries initiating screening programs, where local epidemiology, available resources, and follow‐up capacity should guide selection. Our findings highlight the diagnostic trade‐offs inherent to each strategy, supporting the adoption of risk‐based algorithms [16, 17, 18].

Genotyping‐alone strategies may be preferable in settings where loss in follow‐up risk is high and triage with reflex secondary tests is not feasible. Extended genotyping can serve as a first‐line tool for risk stratification, guiding triage and management in the general population and among women living with HIV [9, 10, 19, 20]. Among genotyping‐alone strategies, G3 targets genotypes HPV16/18/45, carrying the highest precancer and cancer progression risks [7, 13, 21], and oncogenic genotypes prevalent in Cameroon, such as HPV31/33/35/52/58. Despite a substantial overtreatment rate, this strategy offers high sensitivity, reducing missed diagnoses and enabling immediate treatment of most CIN3+ disease. Channel 4 (HPV 51/59) and Channel 5 (HPV 39/56/66/68) demonstrated very low absolute risk, consistent with De Marco et al. (< 1% over 7 years of follow‐up) (13), supporting management through follow‐up alone. Improving access to affordable point‐of‐care HPV assays targeting key genotypes will be essential for the widespread implementation of this strategy [22].

If triage with reflex secondary tests is feasible based on local resources and trained staff, specificity can improve, and unnecessary treatment can decrease, complementing the high sensitivity of genotyping. VIA remains a recommended triage method in low‐resource settings owing to its simplicity, low cost, and potential for immediate clinical decision‐making [2]. However, VIA presents a high variability in diagnostic performance [23, 24, 25] because visual interpretation is highly subjective and depends largely on the practitioner's experience, necessitating specialized training [25]. In meta‐analyses, VIA showed a pooled sensitivity ranging from 48% to 83% for CIN2+ lesion detection, while pooled specificity ranged from 84% to 97% when used as a primary screening test [26].

In our analysis, targeted VIA in selected genotypes, such as in GV13 (targeting channels 2 and 3), improved sensitivity to 83.8%. Nevertheless, PPV remained modest at 16.4% (Figure 4). Ongoing studies are evaluating smartphone‐based automated VIA classifiers using artificial intelligence (AI), showing potential for objective identification of precancerous and cancerous lesions, improving VIA accuracy and reproducibility [27, 28, 29]. In recent years, extended genotyping combined with cytology has been increasingly investigated, as this triage combination effectively stratifies immediate and long‐term risk among women with HPV [30]. In our analysis, among the 35 strategies, extended genotyping with reflex cytology provided the best balance between benefits and harms, minimizing both undertreatment and overtreatment. Strategy GC13 demonstrated a high sensitivity of 91.2%, specificity of 76.0%, and PPV of 25.2% for CIN3+ detection (Figure 4).

Cytology performed better across most strategies compared to VIA, particularly when used to triage intermediate‐risk groups such as channel 2‐ or 3‐positive women. Cytology may be particularly relevant for triaging HPV18/45‐positive women, given their increased risk of adenocarcinoma [31]. However, skilled cytopathologists are scarce in many LMICs [2]. Potential solutions to high‐quality cytology screening include the introduction of new technologies, such as tele‐cytology and AI‐assisted cytodiagnosis [32, 33, 34]. Studies in Cameroon are currently evaluating the feasibility and cost‐effectiveness of these technologies for triage of women with HPV [35].

This study demonstrated the relevance of hierarchically grouping oncogenic HPV genotypes into four categories for differential risk‐based management. The absolute risk of ≥ CIN3 for HPV16 was 32.2%; for HPV18/45, 9.8%; for HPV31/33/35/52/58, 6.4%; and for HPV51/59/39/56/66/68, < 3%. These results are in line with the recent systematic analysis of the world literature by the IARC [7]. Therefore, proposing an effective triage for LMICs and planning management by stratifying women's baseline risk for a histological endpoint of CIN3+ is feasible.

A strength of our study is the comparison of 35 strategies in a large cohort of 868 HPV‐positive women, using biopsy and ECB results as the gold standard for all HPV‐positive women. While other studies have compared various screening strategies (ATHENA, NTCC2), the strategies included were less extensive [30, 36]. Many analyses were based on modelling [37], and some used cytology as the gold standard [38]. This cohort of 868 HPV‐positive women is also one of the first to use VIA, genotyping, and cytology examination. The 35 strategies are based on real patients' data and not modelling.

However, our screening strategies are based on a single cross‐sectional visit, without follow‐up on disease progression, treatment, or surveillance outcomes. VIA was performed under research conditions with regular training and supervision, and cytology results were interpreted at the University of Geneva with knowledge of HPV status, which may have inflated diagnostic performance. HIV status was self‐reported, which may have led to misclassification. We did not use HIV status as a stratification variable. However, the relatively low proportion of HIV‐positive women in our sample may enhance the generalizability of our findings to the broader screening population, as the HIV prevalence observed (4.6%) was similar to that reported in women aged 25–50 years in Cameroon (5.2%) [39]. In the GeneXpert assay, HPV 18 and 45 are grouped in channel 2, without distinction. This stratification assumes similar risks for HPV 18 and 45, which might not be appropriate [13, 31]. A further limitation is that the study population was restricted to women aged 30–49 years. Although this reflects WHO recommendations for CC screening in LMICs, it may limit the generalizability of our findings to older women, in whom the performance of visual inspection methods may differ.

Future research should assess the use of tele‐cytology and AI to enable cytology‐based triage in low‐resource settings. A randomized trial comparing genotyping alone (e.g., G3) versus genotyping plus cytology (e.g., GC13) could clarify clinical performance and acceptability in real life, particularly regarding direct treatment of HPV‐positive women and potential loss to follow‐up due to delayed cytological results. HPV tests that distinguish HPV18 from HPV45 should also be evaluated, considering their potentially different risk profiles. Risk models integrating additional factors (e.g., age, HIV status) may further guide management decisions.

## 5 Conclusion

This study compared 35 screening and triage strategies to manage HPV‐positive women, combining HPV genotyping alone or with VIA or cytology, and considering different options for treatment, triage, or follow‐up. We propose three practical strategies adapted to resource availability: (1) HPV extended genotyping alone, (2) genotyping and VIA, and (3) genotyping and cytology. Overall, strategies combining extended genotyping and cytology performed best regarding sensitivity and specificity. If no triage with VIA or cytology is available, extended genotyping could guide treatment and eight highest‐risk HPV types (16/18/45/31/33/35/52/58) should be considered. Where cytology or VIA is available, HPV16‐positive women should be treated directly. Women positive for HPV18/45/31/33/35/52/58 should undergo reflex triage, either by cytology (treating ≥ ASCUS lesions) or VIA. Women with isolated HPV51/59 or HPV39/56/66/68 should be followed up without immediate treatment. Combining HPV genotyping with cytology or VIA enables risk‐adapted triage and guides management decisions based on available resources. These findings may guide the selection of effective and context‐appropriate triage strategies within screen‐triage‐treat approaches in LMICs.

## Author Contributions

**Micol Murtas:** conceptualization, methodology, data curation, formal analysis, funding acquisition, writing – original draft, writing – review and editing, validation, visualization. **Pierre Vassilakos:** conceptualization, methodology, supervision, visualization, writing – review and editing. **Ania Wisniak:** visualization, validation, methodology, funding acquisition, conceptualization, writing – review and editing, data curation. **Ketty Hu:** writing – review and editing, visualization, methodology. **Marc Arbyn:** methodology, visualization, writing – review and editing, conceptualization. **Gilles W. Tankeu Happi:** investigation, resources, data curation, writing – review and editing. **Esther Abatsong:** writing – review and editing, investigation, resources. **François Marcel Ndam Nsangou:** writing – review and editing, investigation, resources. **Jean‐Christophe Tille:** writing – review and editing, investigation, validation. **Essia Saiji:** writing – review and editing, investigation, validation. **Bruno Kenfack:** writing – review and editing, investigation, project administration, supervision. **Patrick Petignat:** writing – review and editing, conceptualization, project administration, resources, funding acquisition, methodology, supervision.

## Funding

This work was supported by the BERC Scholarship (University of Geneva) and Swiss Cancer Research (KFS 5312‐02‐2021). MA received support from the European Commission Initiative on Cervical Cancer (CE‐CvC), the EUCanScreen Joint Action (grant 101162959) funded by the EU4Health Programme of the European Commission, and the World Health Organisation (through the Agreement for Performance of Work for Guidelines on Screening and Treatment of Pre‐invasive Cervical Disease). The funders had no role in the study design, data collection, analysis, or interpretation, nor in the writing of the manuscript or the decision to publish the findings.

## Ethics Statement

The 3T study was approved by the Cantonal Ethics Committee of Geneva 2017–01110 and the National Ethics Committee for Human Research in Cameroon No. 2018/07/1083/CE/CNERSH/SP. The 3T study is registered at ClinicalTrials.gov (NCT03757299). All participants provided written informed consent.

## Conflicts of Interest

The authors declare no conflicts of interest.

## Supporting information


**Table S1:** Sociodemographic characteristics of HPV‐positive and HPV‐negative participants.
**Table S2:** HIV status among the included population and HPV‐positive participants, and HPV status among participants living with HIV.
**Table S3:** Distribution of HPV channels, cytology, and VIA according to histological results.
**Table S4:** Diagnostic performance, treatment burden, and overtreatment of triage strategies for CIN2+ detection.
**Table S5:** Pairwise comparison of sensitivity and specificity between selected screening strategies using McNemar's test.
**Figure S1:** Diagram showing the 3T study algorithm for treatment and data collection.
**Figure S2:** Receiver operating characteristic curve analysis and efficient frontier among the 35 triage strategies.
**Figure S3:** Comparison of PPV, cNPV, and overtreatment rates for CIN2+ and CIN3+ detection across cervical screening strategies.
**Figure S4:** Preferred strategies for CIN2+ detection according to available resources.

## Data Availability

Deidentified participant data and the corresponding data dictionary are available from the corresponding author upon reasonable request, after approval of a proposal and a data access agreement.

## References

[1] J. Wu , Q. Jin , Y. Zhang , et al., “Global Burden of Cervical Cancer: Current Estimates, Temporal Trend and Future Projections Based on the GLOBOCAN 2022,” Journal of the National Cancer Center 5, no. 3 (2025): 322–329, https://www.sciencedirect.com/science/article/pii/S2667005425000134. 40693230 10.1016/j.jncc.2024.11.006 PMC12276544

[2] Organisation Mondiale de la Santé (OMS) , “Target Product Profiles for Human Papillomavirus Screening Tests to Detect Cervical Pre‐Cancer and Cancer [En Ligne],” 2024, https://iris.who.int/bitstream/handle/10665/379099/9789240100275‐eng.pdf?sequence=1.

[3] “New Recommendations for Screening and Treatment to Prevent Cervical Cancer [Internet],” 2025, https://www.who.int/news/item/06‐07‐2021‐new‐recommendations‐for‐screening‐and‐treatment‐to‐prevent‐cervical‐cancer.

[4] T. Brandt , S. B. Wubneh , S. Handebo , et al., “Genital Self‐Sampling for HPV‐Based Cervical Cancer Screening: A Qualitative Study of Preferences and Barriers in Rural Ethiopia,” BMC Public Health 19 (2019): 1026. 31366402 10.1186/s12889-019-7354-4 PMC6669971

[5] N. G. Campos , M. Mvundura , J. Jeronimo , F. Holme , E. Vodicka , and J. J. Kim , “Cost‐Effectiveness of HPV‐Based Cervical Cancer Screening in the Public Health System in Nicaragua,” BMJ Open 7 (2017): e015048. 10.1136/bmjopen-2016-015048 PMC5623348 28619772

[6] R. Sankaranarayanan , “Screening for Cancer in Low‐ and Middle‐Income Countries,” Annals of Global Health 80 (2014): 412–417. 25512156 10.1016/j.aogh.2014.09.014

[7] F. Wei , D. Georges , I. Man , I. Baussano , and G. M. Clifford , “Causal Attribution of Human Papillomavirus Genotypes to Invasive Cervical Cancer Worldwide: A Systematic Analysis of the Global Literature,” Lancet 404 (2024): 435–444. 39097395 10.1016/S0140-6736(24)01097-3

[8] M. Pinheiro , J. C. Gage , G. M. Clifford , et al., “Association of HPV35 With Cervical Carcinogenesis Among Women of African Ancestry: Evidence of Viral‐Host Interaction With Implications for Disease Intervention [Internet],” International Journal of Cancer 147, no. 10 (2020): 2677–2686, https://pubmed.ncbi.nlm.nih.gov/32363580/. 32363580 10.1002/ijc.33033 PMC11090644

[9] C. Broquet , P. Vassilakos , F. M. N. Nsangou , et al., “Utility of Extended HPV Genotyping for the Triage of Self‐Sampled HPV‐Positive Women in a Screen‐And‐Treat Strategy for Cervical Cancer Prevention in Cameroon: A Prospective Study of Diagnostic Accuracy [Internet],” BMJ Open 12, no. 12 (2022): e057234, https://pubmed.ncbi.nlm.nih.gov/36549727/. 10.1136/bmjopen-2021-057234 PMC9791451 36549727

[10] L. Kuhn , R. Saidu , R. Boa , et al., “Clinical Evaluation of Modifications to a Human Papillomavirus Assay to Optimise Its Utility for Cervical Cancer Screening in Low‐Resource Settings: A Diagnostic Accuracy Study,” Lancet Global Health 8 (2020): e296–e304. 31981559 10.1016/S2214-109X(19)30527-3 PMC8720235

[11] J. Levy , M. de Preux , B. Kenfack , et al., “Implementing the 3T‐Approach for Cervical Cancer Screening in Cameroon: Preliminary Results on Program Performance,” Cancer Medicine 9 (2020): 7293–7300. 32757469 10.1002/cam4.3355 PMC7541141

[12] “ABCD Criteria to Improve Visual Inspection With Acetic Acid (VIA) Triage in HPV‐Positive Women: A Prospective Study of Diagnostic Accuracy,” 2025, https://pubmed.ncbi.nlm.nih.gov/35379615/. 10.1136/bmjopen-2021-052504 PMC8981272 35379615

[13] M. Demarco , N. Hyun , O. Carter‐Pokras , et al., “A Study of Type‐Specific HPV Natural History and Implications for Contemporary Cervical Cancer Screening Programs [Internet],” EClinicalMedicine 22 (2020): 100293, https://pubmed.ncbi.nlm.nih.gov/32510043/. 32510043 10.1016/j.eclinm.2020.100293 PMC7264956

[14] K. R. Kroon , J. A. Bogaards , D. A. M. Heideman , et al., “Colposcopy Referral and CIN3+ Risk of Human Papillomavirus Genotyping Strategies in Cervical Cancer Screening,” Cancer Epidemiology, Biomarkers & Prevention 33, no. 8 (2024): 1037–1045, https://pubmed.ncbi.nlm.nih.gov/38546394/. 10.1158/1055-9965.EPI-24-0046 38546394

[15] H. Markowitz , “Portfolio Selection,” Journal of Finance 7 (1952): 77–91.

[16] R. B. Perkins , D. L. Smith , J. Jeronimo , et al., “Use of Risk‐Based Cervical Screening Programs in Resource‐Limited Settings,” Cancer Epidemiology 84 (2023): 102369. 37105017 10.1016/j.canep.2023.102369

[17] N. Wentzensen , M. Schiffman , T. Palmer , and M. Arbyn , “Triage of HPV Positive Women in Cervical Cancer Screening,” Journal of Clinical Microbiology 76, no. Suppl 1 (2016): S49–S55. 10.1016/j.jcv.2015.11.015 PMC4789103 26643050

[18] R. B. Perkins , R. S. Guido , P. E. Castle , et al., “2019 ASCCP Risk‐Based Management Consensus Guidelines: Updates Through 2023,” Journal of Lower Genital Tract Disease 28 (2024): 3–6. 38117563 10.1097/LGT.0000000000000788 PMC10755815

[19] M. Schiffman , M. J. Khan , D. Solomon , et al., “A Study of the Impact of Adding HPV Types to Cervical Cancer Screening and Triage Tests,” Journal of the National Cancer Institute 97 (2005): 147–150. 15657345 10.1093/jnci/dji014

[20] J. Cuzick and C. Wheeler , “Need for Expanded HPV Genotyping for Screening,” Papillomavirus Research 2 (2016): 2–115. 10.1016/j.pvr.2016.05.004 PMC5886893 29074170

[21] P. Guan , R. Howell‐Jones , N. Li , et al., “Human Papillomavirus Types in 115,789 HPV‐Positive Women: A Meta‐Analysis From Cervical Infection to Cancer,” International Journal of Cancer 131 (2012): 2349–2359. 22323075 10.1002/ijc.27485

[22] J. Wang , G. Imade , A. S. Akanmu , et al., “Analytical Performance of the ScreenFire HPV RS Zebra BioDome Assay on Four Different qPCR Platforms,” Infectious Agents and Cancer 20 (2025): 28. 40307888 10.1186/s13027-025-00651-5 PMC12042583

[23] P. Debeaudrap , F. N. Kabore , L. Setha , et al., “Performance of Visual Inspection, Partial Genotyping, and Their Combination for the Triage of Women Living With HIV Who Are Screen Positive for Human Papillomavirus: Results From the AIMA‐CC ANRS 12375 Multicentric Screening Study [Internet],” International Journal of Cancer 156, no. 3 (2025): 598–607, https://pubmed.ncbi.nlm.nih.gov/39319557/. 39319557 10.1002/ijc.35190 PMC11621995

[24] M. Almonte , C. Ferreccio , S. Luciani , et al., “Visual Inspection After Acetic Acid (VIA) is Highly Heterogeneous in Primary Cervical Screening in Amazonian Peru,” PLoS One 10 (2015): e0115355. 25635965 10.1371/journal.pone.0115355 PMC4312028

[25] A. Baena , D. Mesher , Y. Salgado , et al., “Performance of Visual Inspection of the Cervix With Acetic Acid (VIA) for Triage of HPV Screen‐Positive Women: Results From the ESTAMPA Study,” International Journal of Cancer 152 (2023): 1581–1592. 36451311 10.1002/ijc.34384 PMC10107773

[26] IARC , “Cervical Cancer Screening [Internet],” 2025, https://publications.iarc.fr/Book‐And‐Report‐Series/Iarc‐Handbooks‐Of‐Cancer‐Prevention/Cervical‐Cancer‐Screening‐2022.

[27] I. Baleydier , P. Vassilakos , R. Viñals , et al., “Study Protocol for a Two‐Site Clinical Trial to Validate a Smartphone‐Based Artificial Intelligence Classifier Identifying Cervical Precancer and Cancer in HPV‐Positive Women in Cameroon,” PLoS One 16 (2021): e0260776. 34914727 10.1371/journal.pone.0260776 PMC8675688

[28] Z. Xue , A. P. Novetsky , M. H. Einstein , et al., “A Demonstration of Automated Visual Evaluation of Cervical Images Taken With a Smartphone Camera,” International Journal of Cancer 147 (2020): 2416–2423. 32356305 10.1002/ijc.33029 PMC11977665

[29] S. de Sanjosé , R. B. Perkins , N. Campos , et al., “Design of the HPV‐Automated Visual Evaluation (PAVE) Study: Validating a Novel Cervical Screening Strategy,” eLife 12 (2024): RP91469. 38224340 10.7554/eLife.91469 PMC10945624

[30] M. Benevolo , G. Ronco , P. Mancuso , et al., “Comparison of HPV‐Positive Triage Strategies Combining Extended Genotyping With Cytology or p16/ki67 Dual Staining in the Italian NTCC2 Study,” eBioMedicine [Internet] 104 (2024): 105149, https://www.thelancet.com/journals/ebiom/article/PIIS2352‐3964(24)00184‐1/abstract. 38759278 10.1016/j.ebiom.2024.105149 PMC11126882

[31] G. M. Clifford , J. S. Smith , M. Plummer , N. Muñoz , and S. Franceschi , “Human Papillomavirus Types in Invasive Cervical Cancer Worldwide: A Meta‐Analysis,” British Journal of Cancer 88 (2003): 63–73. 12556961 10.1038/sj.bjc.6600688 PMC2376782

[32] P. Vassilakos , H. Clarke , M. Murtas , et al., “Telecytologic Diagnosis of Cervical Smears for Triage of Self‐Sampled Human Papillomavirus‐Positive Women in a Resource‐Limited Setting: Concept Development Before Implementation [Internet],” Journal of the American Society of Cytopathology 12, no. 3 (2023): 170–180, https://pubmed.ncbi.nlm.nih.gov/36922319/. 36922319 10.1016/j.jasc.2023.02.001

[33] O. Holmström , N. Linder , H. Kaingu , et al., “Point‐Of‐Care Digital Cytology With Artificial Intelligence for Cervical Cancer Screening in a Resource‐Limited Setting,” JAMA Network Open 4 (2021): e211740. 33729503 10.1001/jamanetworkopen.2021.1740 PMC7970338

[34] T. Stegmüller , C. Abbet , B. Bozorgtabar , et al., “Self‐Supervised Learning‐Based Cervical Cytology for the Triage of HPV‐Positive Women in Resource‐Limited Settings and Low‐Data Regime,” Computers in Biology and Medicine 169 (2024): 107809. 38113684 10.1016/j.compbiomed.2023.107809

[35] E. d. Weck , P. Vassilakos , A. Wisniak , M. Murtas , P. Petignat , and G. E. Orock , “Implementation of Point‐Of‐Care Cytology for the Triage of Human Papillomavirus‐Positive Women in Cameroon,” International Journal of Gynecological Cancer 35, no. 10 (2025): 101840, https://www.international‐journal‐of‐gynecological‐cancer.com/article/S1048‐891X(25)00959‐4/abstract. 39242101 10.1136/ijgc-2024-005849

[36] P. E. Castle , M. H. Stoler , T. C. Wright , A. Sharma , T. L. Wright , and C. M. Behrens , “Performance of Carcinogenic Human Papillomavirus (HPV) Testing and HPV16 or HPV18 Genotyping for Cervical Cancer Screening of Women Aged 25 Years and Older: A Subanalysis of the ATHENA Study,” Lancet Oncology 12 (2011): 880–890. 21865084 10.1016/S1470-2045(11)70188-7

[37] K. T. Simms , A. Keane , D. T. N. Nguyen , et al., “Benefits, Harms and Cost‐Effectiveness of Cervical Screening, Triage and Treatment Strategies for Women in the General Population,” Nature Medicine 29 (2023): 3050–3058. 10.1038/s41591-023-02600-4 PMC10719104 38087115

[38] A. J. B. Vallely , M. Saville , S. G. Badman , et al., “Point‐Of‐Care HPV DNA Testing of Self‐Collected Specimens and Same‐Day Thermal Ablation for the Early Detection and Treatment of Cervical Pre‐Cancer in Women in Papua New Guinea: A Prospective, Single‐Arm Intervention Trial (HPV‐STAT),” Lancet Global Health 10 (2022): e1336–e1346. 35878625 10.1016/S2214-109X(22)00271-6

[39] National Institute of Statistics (Cameroon) and ICF , 2018 Cameroon DHS Summary Report (NIS and ICF [Internet], 2020), https://dhsprogram.com/pubs/pdf/SR266/SR266.pdf.

