# Supplementary material for: Resource‐Adapted Triage Strategies for Women Testing HPV Positive With Self‐Collected Vaginal Samples in Cameroon
Source: Int J Cancer. 2026 Jun 18;159(8):1949–61. doi: 10.1002/ijc.70573 (PMC13495884; doi:10.1002/ijc.70573)
Supplement: Supplementary file 1 [file IJC-159-1949-s001.pdf]

Resource-adapted triage strategies for women testing HPV positive with self-collected vaginal samples in Cameroon

Micol Murtas, Pierre Vassilakos, Ania Wisniak, Ketty Hu, Marc Arbyn, Gilles W. Tankeu Happi, Esther Abatsong, François Marcel Ndam Nsangou, Jean-Christophe Tille, Essia Saiji, Bruno Kenfack, Patrick Petignat

Table of content

Table S1. Sociodemographic characteristics of HPV-positive and HPV-negative participants

Table S2. HIV status among the included population and HPV-positive participants, and HPV status among participants living with HIV

Table S3. Distribution of HPV channels, cytology, and VIA according to histological results

Table S4. Diagnostic performance, treatment burden, and overtreatment of triage strategies for CIN2+ detection

Table S5. Pairwise comparison of sensitivity and specificity between selected screening strategies using McNemar's test

Figure S1. Diagram showing the 3T study algorithm for treatment and data collection

Figure S2. Receiver operating characteristic curve analysis and efficient frontier among the 35 triage strategies

Figure S3. Comparison of PPV, cNPV, and overtreatment rates for CIN2+ and CIN3+ detection across cervical screening strategies

Figure S4. Preferred strategies for CIN2+ detection according to available resources

Supplementary tables

Table S1: Sociodemographic characteristics of HPV-positive and negative participants

	Total population (4424) N (%)	HPV + (868) N (%)	HPV - (3554) N (%)	Valid data N (%)
**Age, mean (SD)**	39.7 (6.1)	39.2 (6.1)	39.8 (6)	4417 (99.8)
**Marital status**				4415 (99.8)
Single	372 (8.4)	100 (11.5)	272 (7.7)	
Inarelationship	1927 (43.6)	384 (44.3)	1543 (43.5)	
Married	1802 (40.8)	297 (34.3)	1505 (42.4)	
Divorced/Widowed	314 (7.1)	86 (9.9)	228 (6.4)	
**Education**				4413 (99.8)
Uneducated	28 (0.6)	10 (1.2)	18 (0.5)	
Primary	1258 (28.5)	247 (28.5)	1011 (28.5)	
Highschool	2306 (52.3)	426 (49.2)	1880 (53.0)	
University	817 (18.5)	182 (21.0)	635 (17.9)	
Other	4 (0.1)	1 (0.1)	3 (0.1)	
**Employment status**				4413 (99.8)
Housewife	815 (18.5)	132 (15.2)	683 (19.3)	
Farmer	1035 (23.5)	214 (24.7)	821 (23.2)	
Employee without responsibility	1028 (23.3)	209 (24.1)	819 (23.1)	
Employee with responsibility	160 (3.6)	32 (3.7)	128 (3.6)	
Self-employed	1224 (27.7)	238 (27.5)	986 (27.8)	
Other	151 (3.4)	42 (4.8)	109 (3.1)	
**Age of menarche, mean (SD)**	14.6 (1.8)	14.6 (1.9)	14.6 (1.8)	4415 (99.8)
**Age at first intercourse, mean (SD)**	18 (2.7)	18 (2.7)	18 (2.7)	4411 (99.7)
**Number of sexual partners, mean (SD)**	3.7 (3.4)	4.1 (3.9)	3.6 (3.3)	4413 (99.8)
**Number of pregnancy, mean (SD)**	5.5 (2.4)	5.1 (2.5)	5.6 (2.4)	4415 (99.8)
**Contraception**				4408 (99.6)
None	3254 (73.8)	640 (74.2)	2614 (73.7)	
Yes	1154 (26.2)	223 (25.8)	931 (26.3)	
IUD	227 (19.8)	38 (17.2)	189 (20.4)	
Condom	440 (38.3)	88 (39.8)	352 (38.0)	
Implant	108 (9.4)	27 (12.2)	81 (8.7)	
Injectable	43 (3.7)	10 (4.5)	33 (3.6)	
Pill	294 (25.6)	53 (24.0)	241 (26.0)	
Other (sterilisation, spermicide)	36 (3.1)	5 (2.3)	31 (3.3)	
**Smoker**				4413 (99.8)
No	4240 (96.1)	835 (96.3)	3405 (96.0)	
Yes	173 (3.9)	32 (3.7)	141 (4.0)	
**HIV status (self-reported)**				4350 (98.3)
Negative	4152 (95.4)	785 (92.5)	3367 (96.2)	

Positive	198 (4.6)	64 (7.5)	134 (3.8)

HPV, human papillomavirus; IUD, intrauterine device.

Table S2: HIV status among the included population and HPV-positive patients. HPV-status among patients with HIV

**HIV-status among included population (4424)**	Values N%	Valid (% of Valid)	Missing
Negative	4152 (95.4%)	4350 (98.3%)	74 (1.7%)
Positive	198 (4.6%)		
**HIV-status among HPV-positive patients (858)**			
Negative	777 (92.6%)	839 (97.8%)	19 (2.2%)
Positive	62 (7.4%)		
**HPV-status among HIV-positive patients (199)**			
Negative	34 (67.7%)	198 (99.5%)	1 (0.5%)
Positive	64 (32.3%)		

Table S3: Distribution of HPV channels, cytology, and VIA by histological results

	**Negative (N=658)**	**CIN 1 (N=95)**	**CIN 2+ (N=37)**	**CIN 3+ (N=57)**	**Cancer (N=11)**	**Total (N=858)**
**Genotyping**						
Channel 1 (HPV 16)	49 (7.4%)	5 (5.3%)	5 (13.5%)	22 (38.6%)	6 (54.5%)	87 (10.1%)
Channel 2 (HPV18/45)	90 (13.7%)	16 (16.8%)	5 (13.5%)	9 (15.8%)	3 (27.3%)	123 (14.3%)
Channel 3 (HPV 31/33/35/52/58)	301 (45.7%)	42 (44.2%)	23 (62.2%)	23 (40.4%)	2 (18.2%)	391 (45.6%)
Channel 4 (HPV 51/59)	68 (10.3%)	13 (13.7%)	1 (2.7%)	1 (1.8%)	0 (0.0%)	83 (9.7%)
Channel 5 (HPV 39/56/66/68)	150 (22.8%)	19 (20.0%)	3 (8.1%)	2 (3.5%)	0 (0.0%)	174 (20.3%)
**Cytology**						
N-Miss	33	0	1	1	2	37
Negative	504 (80.6%)	59 (62.1%)	7 (19.4%)	8 (14.3%)	0 (0.0%)	578 (70.4%)
Positive (≥ ASCUS)	121 (19.4%)	36 (37.9%)	29 (80.6%)	48 (85.7%)	9 (100.0%)	243 (29.6%)
**VIA**						
N-Miss	2	0	0	0	0	2
Negative	353 (53.8%)	27 (28.4%)	13 (35.1%)	10 (17.5%)	3 (27.3%)	406 (47.4%)
Positive	303 (46.2%)	68 (71.6%)	24 (64.9%)	47 (82.5%)	8 (72.7%)	450 (52.6%)

VIA, Visual inspection assessment; CIN2+, cervical intraepithelial neoplasia grade three or worse; CIN3+, cervical intraepithelial neoplasia grade three or worse; ASC-US, atypical squamous cells of undetermined significance or worse;

Table S4: Sensitivity, specificity, PPV, NPV, women treated per CIN2+ diagnosed, and overtreatment of triage strategies.

	Sensitivity	Specificity	PPV	cNPV	Overtreatment in HPV + population	Women treated per CIN2+ diagnosed	Overtreatment in screened population
G 1*	100.0 (96.5-100.0)	0.0 (0.0-0.5)	12.2 (10.1-14.6)	NA (1-0)	87.8 (85.4-89.9)	8.17 (7.62-8.72)	17.1 (16.0-18.2)
G 2	95.2 (89.2-98.4)	22.4 (19.5-25.6)	14.6 (12.1-17.5)	2.9 (0.9-6.6)	85.4 (82.5-87.9)	6.84 (6.33-7.35)	13.2 (12.3-14.3)
G 3	93.3 (86.7-97.3)	33.2 (29.8-36.7)	16.3 (13.4-19.5)	2.7 (1.1-5.5)	83.7 (80.5-86.6)	6.13 (5.64-6.62)	11.4 (10.5-12.4)
G 4	47.6 (37.8-57.6)	78.8 (75.7-81.6)	23.8 (18.2-30.2)	8.5 (6.5-10.9)	76.2 (69.8-81.8)	4.20 (3.63-4.77)	3.6 (3.1-4.2)
G 5	31.4 (22.7-41.2)	92.8 (90.7-94.6)	37.9 (27.7-49.0)	9.3 (7.4-11.6)	62.1 (51.0-72.3)	2.64 (2.08-3.19)	1.2 (0.9-1.6)
**Genotyping with triage by VIA**							
GV 1*	75.2 (65.9-83.1)	50.6 (47.0-54.2)	17.6 (14.2-21.4)	6.4 (4.2 – 9.2)	82.4 (78.6-85.8)	5.70 (5.17-6.22)	8.4 (7.6-9.3)
GV 2	72.4 (62.8-80.7)	62.6 (59.1-66.1)	21.3 (17.2-25.9)	5.8 (3.9 – 8.2)	78.7 (74.1-82.8)	4.70 (2.17-2.84)	6.4 (5.7-7.1)
GV 3	70.5 (60.8-79.0)	67.8 (64.3-71.1)	23.4 (18.9-28.5)	5.7 (3.9 – 8.0)	76.6 (71.5-81.1)	4.27 (3.80-4.74)	5.5 (4.8-6.2)
GV 4	40.0 (30.6-50.0)	90.0 (87.7-92.1)	35.9 (27.2-45.3)	8.5 (6.6 – 10.8)	64.1 (54.7-72.8)	2.79 (2.28-3.29)	1.7 (1.3-2.1)
GV 5	27.6 (19.3-37.2)	96.4 (94.8-97.6)	51.8 (38.0-65.3)	9.5 (7.5 – 11.7)	48.2 (34.7-62.0)	1.93 (1.43-2.44)	0.6 (0.4-0.9)
GV 6	79.0 (70.0-86.4)	47.0 (43.4-50.6)	17.3 (14.0-20.9)	5.9 (3.7 – 8.7)	82.7 (79.1-86.0)	5.80 (5.28-6.31)	9.0 (8.2-9.9)
GV 7*	82.9 (74.3-89.5)	39.4 (35.9-43.0)	16.0 (13.0-19.4)	5.7 (3.4 – 8.9)	84.0 (80.6-87.0)	6.24 (5.72-6.77)	10.3 (9.5-11.3)
GV 8	98.1 (93.3-99.8)	16.0 (13.4-18.8)	14.0 (11.6-16.7)	1.6 (0.2 – 5.8)	86.0 (83.3-88.4)	7.14 (6.62-7.65)	14.3 (13.3-15.4)
GV 9	98.1 (93.3-99.8)	10.4 (8.3-12.8)	13.3 (11.0-15.8)	2.5 (0.3 – 8.7)	86.7 (84.2-89.0)	7.54 (7.01-8.07)	15.3 (14.2-16.4)
GV 10	76.2 (66.9-84.0)	59.0 (55.4-62.6)	20.6 (16.7-25.0)	5.3 (3.5 – 7.8)	79.4 (75.0-83.3)	4.85 (4.37-5.33)	7.0 (6.3-7.8)
GV 11	80.0 (71.1-87.2)	51.4 (47.8-55.0)	18.7 (15.2-22.6)	5.1 (3.2 – 7.8)	81.3 (77.4-84.8)	5.36 (4.86-5.85)	8.3 (7.5-9.2)
GV 12	95.2 (89.2-98.4)	28.0 (24.8-31.4)	15.6 (12.9-18.6)	2.3 (0.8 – 5.3)	84.4 (81.4-87.1)	6.42 (5.92-6.92)	12.3 (11.3-13.3)
GV 13	74.3 (64.8-82.3)	64.2 (60.7-67.7)	22.5 (18.2-27.2)	5.3 (3.5 – 7.6)	77.5 (72.8-81.8)	4.45 (3.98-4.92)	6.1 (5.4-6.9)
GV 14	78.1 (69.0-85.6)	56.6 (52.9-60.1)	20.0 (16.3-24.3)	5.1 (3.3 – 7.6)	80.0 (75.7-83.7)	4.99 (4.50-5.47)	7.4 (6.7-8.2)
GV 15	43.8 (34.1-53.8)	86.4 (83.8-88.8)	31.1 (23.7-39.2)	8.3 (6.4 – 10.6)	68.9 (60.8-76.3)	3.22 (2.70-3.74)	2.3 (1.9-2.8)
**Genotyping with triage by cytology (≥ASCUS)**							
GC 1*	85.1 (76.7-91.4)	78.2 (75.0-81.2)	35.4 (29.4-41.8)	2.6 (1.5 – 4.2)	64.6 (58.2-70.6)	2.83 (2.47-3.18)	3.6 (3.0-4.2)
GC 2	83.3 (74.7-90.0)	82.4 (79.4-85.1)	39.9 (33.3-46.8)	2.8 (1.6 – 4.4)	60.1 (53.2-66.7)	2.51 (2.17-2.84)	2.9 (2.4-3.4)
GC 3	81.4 (72.4-88.4)	84.2 (81.4-86.8)	41.9 (35.0-49.1)	3.0 (1.8 – 4.6)	58.1 (50.9-65.0)	2.39 (2.05-2.72)	2.6 (2.2-3.1)
GC 4	39.2 (29.7-49.4)	95.4 (93.7-96.8)	54.1 (42.1-65.7)	8.0 (6.2 – 10.2)	45.9 (34.3-57.9)	1.85 (1.43-2.27)	0.8 (0.5-1.1)
GC 5	25.2 (17.2-34.8)	98.3 (97.1-99.1)	66.7 (49.8-80.9)	9.4 (7.5 – 11.7)	33.3 (19.1-50.2)	1.50 (1.03-1.97)	0.3 (0.2-0.5)
GC 6	90.3 (82.9-95.2)	72.6 (69.2-75.8)	32.0 (26.6-37.7)	1.9 (0.9 – 3.4)	68.0 (62.3-73.4)	3.13 (2.77-3.49)	4.5 (3.9-5.1)
GC 7	92.3 (85.4-96.6)	61.1 (57.4-64.6)	25.3 (21.0-30.0)	1.8 (0.8 – 3.5)	74.7 (70.0-79.0)	3.95 (3.55-4.35)	6.4 (5.7-7.2)
GC 8	97.1 (91.8-99.4)	26.6 (23.5-30.0)	15.6 (12.9-18.7)	1.5 (0.3 – 4.3)	84.4 (81.3-87.1)	6.40 (5.90-6.89)	12.3 (11.4-13.4)
GC 9	97.1 (91.8-99.4)	17.9 (15.3-20.9)	14.1 (11.7-16.9)	2.2 (0.5 – 6.3)	85.9 (83.1-88.3)	7.07 (6.55-7.59)	13.9 (12.9-15.0)
GC 10	88.5 (80.7-93.9)	76.8 (73.5-79.8)	35.2 (29.5-41.4)	2.1 (1.1 – 3.6)	64.8 (58.6-70.5)	2.84 (2.49-3.18)	3.8 (3.3-4.4)
GC 11	90.5 (83.2-95.3)	65.3 (61.8-68.8)	27.2 (22.6-32.2)	2.0 (1.0 – 3.7)	72.8 (67.8-77.4)	3.67 (3.29-4.06)	5.8 (5.1-6.5)

GC 12	95.2 (89.2-98.4)	31.1 (27.8-34.6)	16.2 (13.4-19.4)	2.1 (0.7 – 4.8)	83.8 (80.6-86.6)	6.16 (5.67-6.65)	11.7 (10.8-12.7)
GC 13	86.5 (78.4-92.4)	78.7 (75.5-81.6)	36.6 (30.6-42.9)	2.4 (1.3 – 3.9)	63.4 (57.1-69.4)	2.73 (2.39-3.07)	3.5 (3.0-4.1)
GC 14	88.6 (80.9-94.0)	67.3 (63.8-70.7)	27.8 (23.1-33.0)	2.4 (1.2 – 4.1)	72.2 (67.0-76.9)	3.59 (3.21-3.98)	5.5 (4.8-6.2)
GC 15	45.2 (35.4-55.3)	90.0 (87.6-92.0)	38.5 (29.9-47.8)	7.8 (6.0 – 10.0)	61.5 (52.2-70.1)	2.60 (2.14-3.06)	1.7 (1.3-2.1)

Channel 1 = HPV16, Channel 2 = HPV18/45, Channel 3 = HPV31/33/35/52/58, Channel 4 = HPV51/59, Channel 5 = HPV39/56/66/68; PPV, positive predictive value; cNPV, complementary negative predictive value; VIA, Visual inspection assessment; ASC-US, atypical squamous cells of undetermined significance or worse; CIN3+, cervical intraepithelial neoplasia grade three or worse; * Recommended strategies in the 2021 WHO report

Table S5: Pairwise comparison of sensitivity and specificity between selected screening strategies using McNemar's test

Comparison	Δ Sensitivity (%)	P-value	Δ Specificity (%)	P-value
G3 vs GC13	4.4	0.248	-43	<0.001
G3 vs GV13	11.8	0.013	-31.1	<0.001
GV13 vs GC13	-7.4	0.182	-12.9	<0.001
GV1 vs GC1	-7.7	0.267	-25	<0.001

Δ indicates the absolute difference between strategies (first minus second). Sensitivity was compared among women with CIN3+, and specificity among women without CIN3+, using McNemar's test for paired proportions. Estimates are based on paired comparisons restricted to women with valid histological results and complete data for the two strategies being compared.

Figure S1 Diagram showing the 3T study algorithm for treatment and collected data

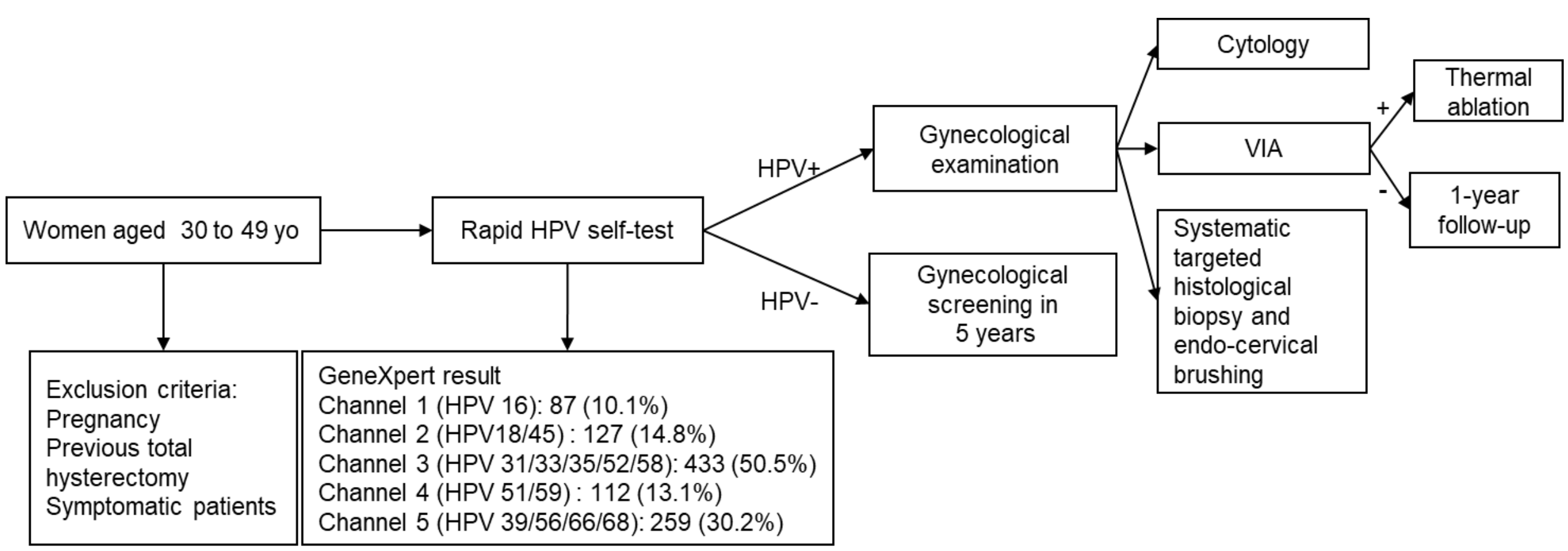

For HPV types, all positive channels are shown without any specific order of severity. VIA, Visual inspection assessment

A **B**

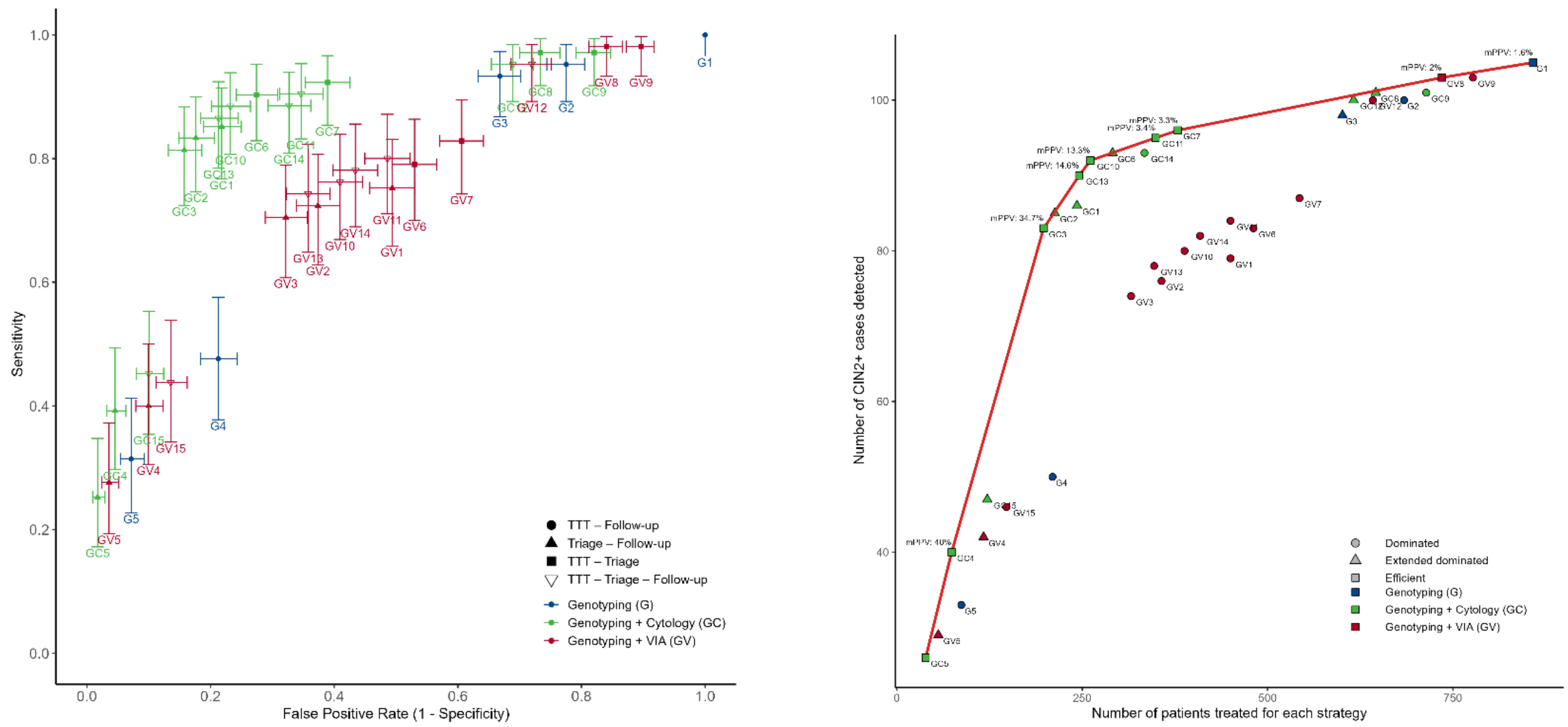

Figure S2: Receiver operating characteristic (ROC) curve analysis (A) and efficient frontier (B) among the 35 triage strategies

A Receiver operating characteristic (ROC) curve analysis of the 35 strategies for detecting cervical intraepithelial neoplasia grade 2 or worse (CIN2+). Strategies combining HPV genotyping, VIA, and cytology are organised around direct treatment, triage, or follow-up.

B Overview of the efficient frontier among the 35 triage strategies. Each strategy is represented by the number of patients testing positive, plotted against the number of CIN2+ cases detected. The red curve highlights the efficient frontier, with annotated points indicating the most efficient strategies and their corresponding marginal positive predictive values (mPPV).

A

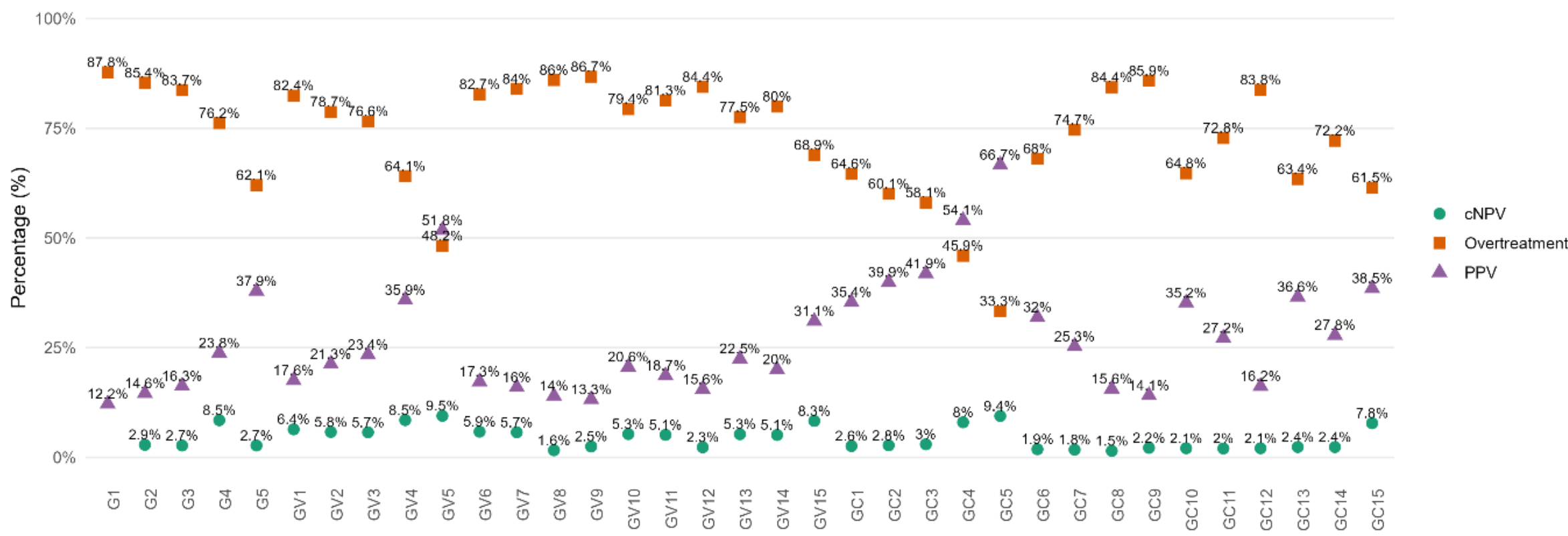

B

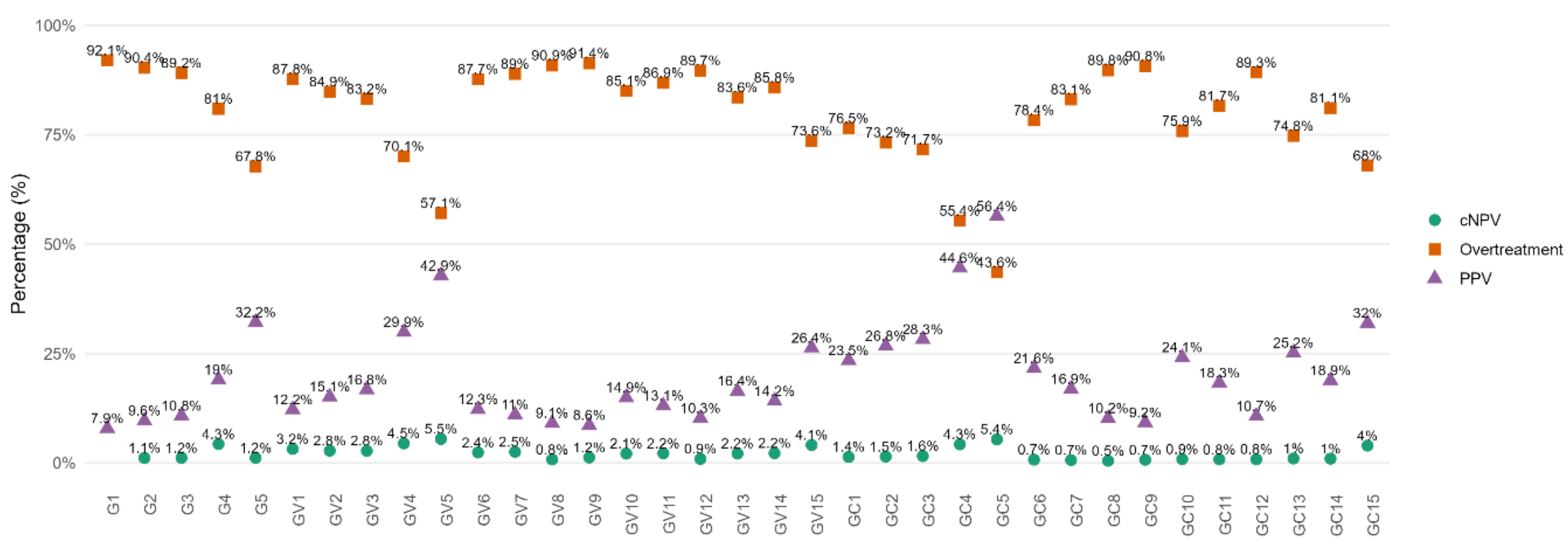

Figure S3 Comparison of PPV, cNPV, and overtreatment rates for CIN2+ (A) and CIN3+ (B) detection across cervical screening strategies.

cNPV, complementary negative predictive value; PPV, positive predictive value

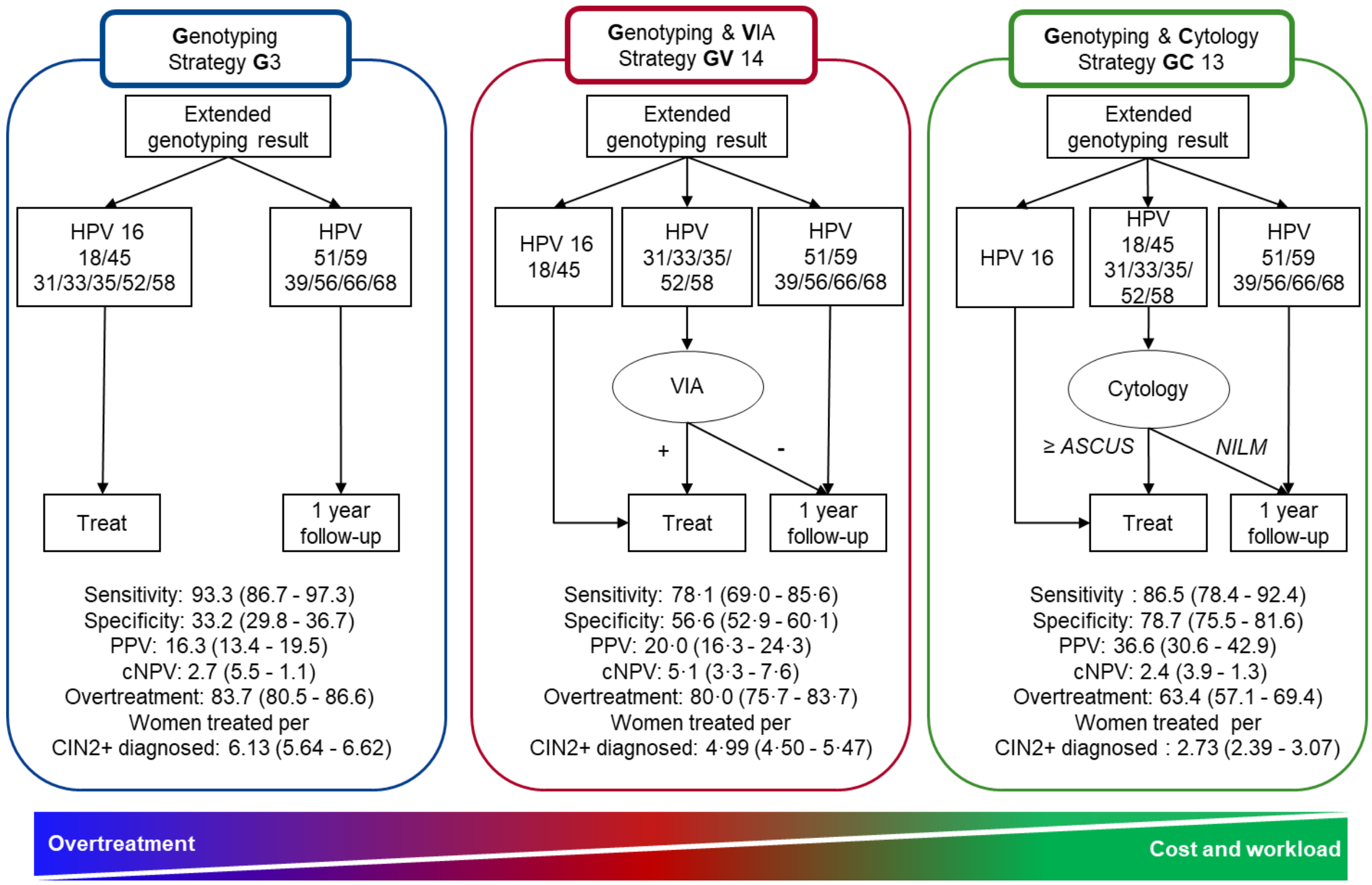

Figure S4: Preferred strategies based on available resources for CIN2+. VIA, Visual inspection assessment.

PPV: positive predictive value; cNPV: complementary negative predictive value; VIA, Visual inspection assessment; ASC-US, atypical squamous cells of undetermined significance or worse; CIN3+, cervical intraepithelial neoplasia grade three or worse
